# Extended phenotypes can underlie trade-offs: a case of social spiders

**DOI:** 10.1007/s00114-022-01826-5

**Published:** 2022-10-29

**Authors:** Bharat Parthasarathy, Michelle Bouchard, Jutta M. Schneider

**Affiliations:** Institute of Cell and Systems Biology of Animals, Universität Hamburg, 20146 Hamburg, Germany

**Keywords:** Animal architecture, Internal state, Foraging safety, Silk, Spider web

## Abstract

Extended phenotypes engineered by animals can potentially improve safety and/or foraging. Whether the well-known trade-off between safety and foraging applies for extended phenotypes, and if so, how it is resolved has not been determined. Spiders build elaborate silk structures that serve as traps for their insect prey and often attach silken retreats (nests) to their capture webs. These extended phenotypes of spiders are made of silk that is considered costly since it is made of protein. Using the Indian social spider, *Stegodyphus sarasinorum*, we examined how simple proximal factors, like colony hunger state and group size, shape trade-offs in collectively built extended phenotypes that offer shelter and food. We found that well-fed colonies showed greater investment in retreat silk than starved colonies. However, the two groups did not differ in their investment in capture webs. Hence, our findings validate the starvation-risk taking hypothesis in an extended phenotypic paradigm by showing that hungry colonies trade-off retreat size for capture web, irrespective of group size.

## Introduction

Many animals build structures such as nests, traps or hides, and such extended phenotypes have long fascinated biologists (Dawkins 1982). Even group living animals such as termites, bees and naked mole rats can collectively engineer impressive architectures that stabilise temperature, facilitate aeration and provide access for food or culturing resources (Wenzel and R. b. 2008). Such animal architectures can significantly alter environmental selection pressures acting on the animals themselves and their offspring in subsequent generations (Clark et al. 2020; Laland et al. 2016). Animals can flexibly adjust the investment in the architecture depending upon the biotic and abiotic environment. For instance, pit-digging *Myrmeleon crudelis* antlions can increase pit diameter when prey is scarce (Farji-Brener and Amador-Vargas 2020). Similarly, hungry *Stegodyphus sarasinorum* spiders invest more in cribellate capture silk than satiated individuals (Ellendula et al. 2021).

Animal architectures can vary within a population (DiRienzo and Aonuma 2018; Walsh et al. 2011), which in turn might explain the stable persistence of alternative behavioural phenotypes within the population. For example, birds that are consistently less aggressive in nest defence (Burtka and Grindstaff 2013; Trnka et al. 2013) might compensate for their potential fitness loss by building more defensive nest structures. Alternatively, investment in a less defensive nest structure might prompt an individual to act more aggressively against potential predators. Therefore, individual behaviour can influence the phenotypes of the animal architecture, and the architectural phenotype in turn can potentially influence behavioural responses of the animal (Montiglio and DiRienzo 2016; Pinter-Wollman 2015). Thus, studies examining behavioural variation in animals exhibiting extended phenotypes are incomplete without integrating their architectural variation in the experimental design.

Extended phenotypes can help animals to forage (e.g. pit traps) and take shelter (e.g. nests). It is well established that animals generally deal with a trade-off between foraging vs. sheltering behaviours (Godin 1986; Lima and Dill 1990; Reaney 2007), and more recent studies demonstrate that the strength of this trade-off depends on the individual’s behavioural type (Farwell and McLaughlin 2009; Steinhoff et al. 2020). However, whether the variation in extended phenotypes within a population itself can contribute to such behavioural trade-offs has received less attention. In this study, we examined the extent to which collectively built web architectures serve primarily as a foraging or sheltering strategy in the Indian social spider, *Stegodyphus sarasinorum*.

*S. sarasinorum* collectively builds a nest (retreat) within which groups of individuals reside and reproduce, and the capture web extends from the entrances of the retreat. The capture web consists of the supporting silken threads and the cribellate silk that captures the prey. Cribellate silk consists of multiple nanofibers that are actively combed out by comb-like structures on the spiders’ hindlegs called the cribellum. On top of the material costs, producing this capture silk entails metabolic costs as the combing is an active process where the spiders rapidly move their hindlegs for extended periods of time (Foelix 2010). The retreat silk is uncombed and not sticky. Whether retreat silk is made of uncombed cribellate or another silk type is currently unknown. We tested the hypothesis that the investment in foraging, measured as capture web size, and in safety, measured as retreat size, underlie the classic trade-off between foraging and sheltering functions. Accordingly, we predicted that hungry colonies would invest relatively more in capture webs and relatively less in retreats than well-fed colonies. Furthermore, we predicted that the per-capita investment in retreat and capture silk would be reduced in larger groups; in comparison to well-fed colonies, starved colonies must show a relatively greater decrease in per-capita retreat investment and a relatively greater increase in cribellate silk investment.

## Methods

### Study organism

*Stegodyphus sarasinorum* is an Indian social spider that collectively builds retreats and capture webs, inbreed with natal kin and show highly female-biased sex ratios (85–90% females). These spiders have low dispersal potential compared to their solitary and subsocial sister species, and therefore, the retreat is likely inherited by a few generations of descendants (Avilés 1997; Lubin and Bilde 2007). However, some individuals leave the main retreat to build satellite retreats at the edges of the capture web. Colonies typically consist of several nests interconnected by common capture webs (Parthasarathy and Somanathan 2018). Solitary dispersal occurs by females after they mated in the natal nest. Solitary dispersers found new colonies.

### Colony collection and construction of experimental colonies

We collected *S. sarasinorum* colonies from Kuppam, Southern India, in January 2021 and exported colonies to Hamburg, Germany, after obtaining the necessary permit from the National Biodiversity Authority of India (permit no: NBA/Tech Appl/9/Form B-152/20/20–21/787). At the University of Hamburg, we maintained colonies at 26 °C, 60% RH and 12 h day-night cycle. In May 2021, we constructed 21 experimental colonies consisting of three different group sizes (1, 10 and 20 spiders) from seven different source colonies by randomly picking subadult kin females. For example, from colony 1, we randomly picked 1, 10 and 20 females to construct experimental colonies 1, 2 and 3, respectively. The group size of the source colonies ranged from 34 to 58 spiders. We housed the females in plastic containers (10 × 6 cm and 3.5 cm high) with mesh lids and gave 2 days to build capture webs inside the boxes so that we could subsequently manipulate colony hunger levels by satiation or starvation. One day before constructing the experimental colonies, we fed the source colonies with ad-libitum blowflies (Calliphoridae) to equalise hunger levels.

### Hunger manipulation and web building

We starved the experimental colonies from the four source colonies for 8 days. With our experience in rearing these spiders, we know that 8 days is sufficient to induce hunger, but not cause death (Parthasarathy et al. 2022). We also housed the experimental colonies from the remaining three source colonies in identical plastic containers for 8 days, but we fed these colonies during the last three consecutive days. We fed colonies with living blowflies (Calliphoridae) abundantly until spiders no longer attacked the fly. The starved and fed colonies were then transferred into individual transparent acrylic frames (36 × 36 × 6 cm deep) covered with mesh and were given 3 days in the dark to build webs. We kept spiders in the dark during this period to facilitate web building, as these spiders are most active under conditions of low light (Jacson and Joseph 1973). On the morning of the fourth day, entire webs along with the retreats, which the spiders built on the top corners of the frames, were photographed under a uniformly dark background using a Sony α-58 (SLT-A58 + Tamron lens 16–300 mm; F/3,5–6,3; diameter 67) camera. The lighting conditions, distance between the camera and the frames consisting colonies, focal length and exposure time of the camera were kept constant for each photograph. We also obtained images from the opposite side of the frame, and from these two images, we calculated the average cribellate area (as described below) for each colony. To examine retreat size, we took photographs from the front and the two sides of the frames. Next, we transferred spiders from the frame belonging to the same experimental group into identical plastic containers and fed the previously starved colonies and starved the previously fed colonies, exactly as described above. As before, we subsequently transferred colonies into transparent acrylic frames and obtained photographs of their webs and retreats.

### Analysing photographs

**Fig. 1:**
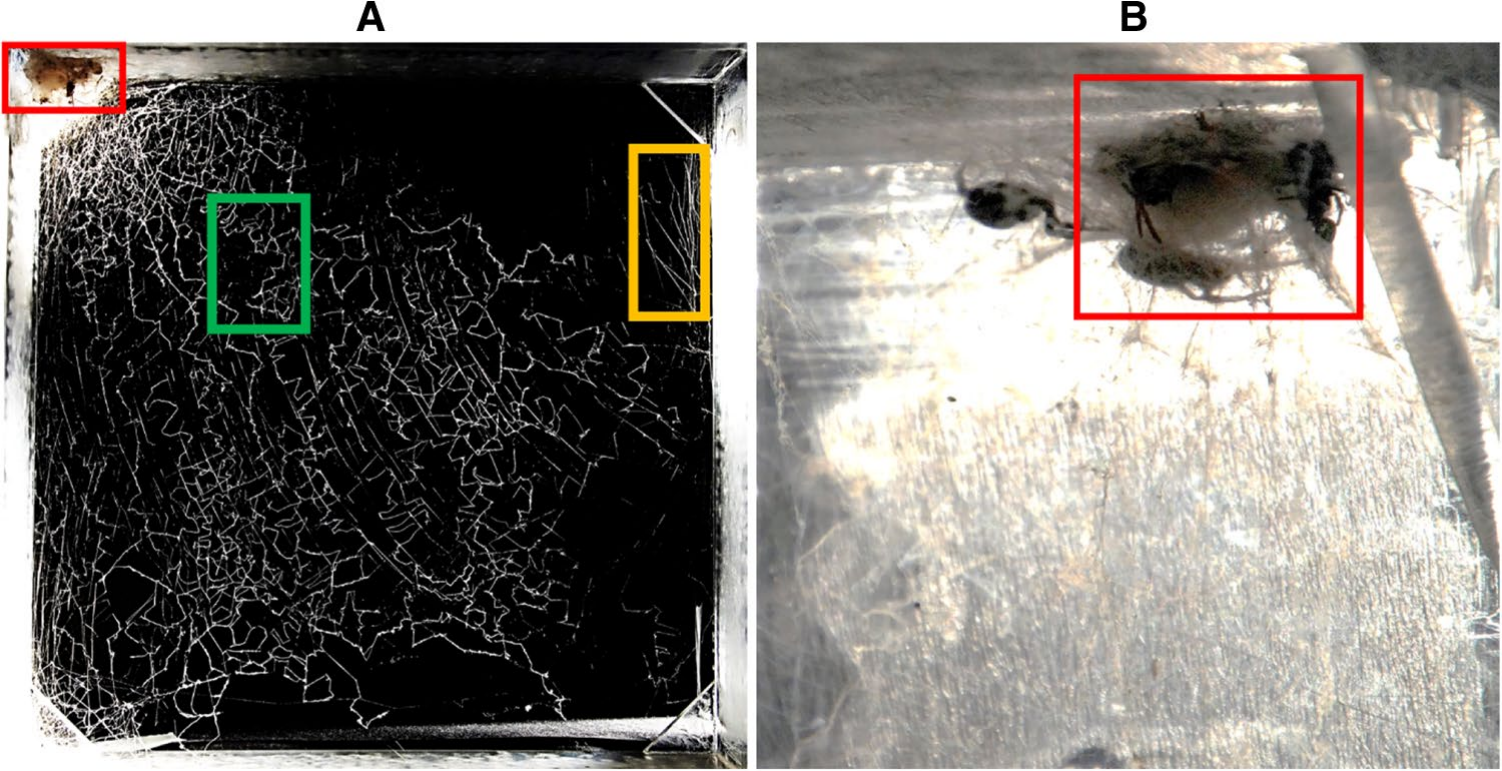
Retreat structure and capture web of *Stegodyphus sarasinorum*. **A**: The retreat is highlighted by a red box. The cribellate silk and supporting silken threads are highlighted by green and yellow boxes, respectively. **B**: Retreat structure viewed from the side of the frame

The photos were reworked with Aurora HDR to make the white web structures more evident. The determination of the web building structures was carried out with ImageJ. We changed the photos into 8-bit type to obtain a black-and-white graphic and removed non-silk sections from the photos. Now, the entire white part (silken part) could be measured. First, the retreat part of the photo and in a second step, the cribellate silk was removed to isolate the area of draglines. Cribellate silk is the combed zig-zag silk (Fig. 1) which traps prey by forming a composite material with the waxy surface of an insect cuticle (Foelix 2010). By using a pixel threshold intensity of 50–255, the area of the silk was marked and measured at every step. Next, the area of cribellate silk could be calculated as the difference between the entire white part and the area of the draglines and the retreat of the photo (see Fig. 1). Because the data from ImageJ was given in pixels, it was necessary to convert the values to mm^2^, using the known area of the frame (1,29,600 mm^2^). The calculation for the retreat volume was based on the formula for a tetrahedron volume $V=\frac{1}{6}*\left|\overrightarrow{a} * \overrightarrow{b}*\overrightarrow{c}\right|$. The required lengths (a, b and c) were measured by a predefined scale in ImageJ.

### Statistical analyses

We performed all statistical analyses using R (v 4.1.0, R core team). First, we estimated per-capita investment in retreat and cribellate silk by dividing the retreat volume and cribellate silk area, respectively, with the group size of colonies. Since we followed a crossed experimental design, we first ruled out the significant interaction effects of order (fed first and starved later or starved first and fed later) with food treatment by building Bayesian mixed models using the ‘rstanarm’ package in R (Goodrich et al. 2020). The per-capita volume of the retreat or the per-capita area of the cribellate silk was the dependent variable, and the interaction terms were food treatment × order. Experimental colony ID and source colony ID were the random effects. As we found no significant interaction between food treatment and order, we could conclude that the order of food treatment administered to colonies did not influence our results on per-capita retreat or cribellate web investment. Next, we built separate Bayesian models for the two dependent variables (per-capita retreat volume or per-capita cribellate area) by including the same random effects as described above, but the interaction term was group size × food treatment. Both group size and food treatment were dummy-coded as categorical variables. We found no significant interaction between food treatment and group size. Therefore, we present the final Bayesian models without this interaction term. The two dependent variables were log transformed to improve model fit. We checked model assumptions by observing the plots consisting of the observed vs. simulated values and diagnosed for chain convergence, chain mixing, divergent transitions and autocorrelation. We ensured that all effective sample sizes were at least > 2000. We also performed Tukey’s post-hoc tests to obtain pairwise contrasts between the two levels of food treatment (well-fed or starved).

## Results

**Fig. 2:**
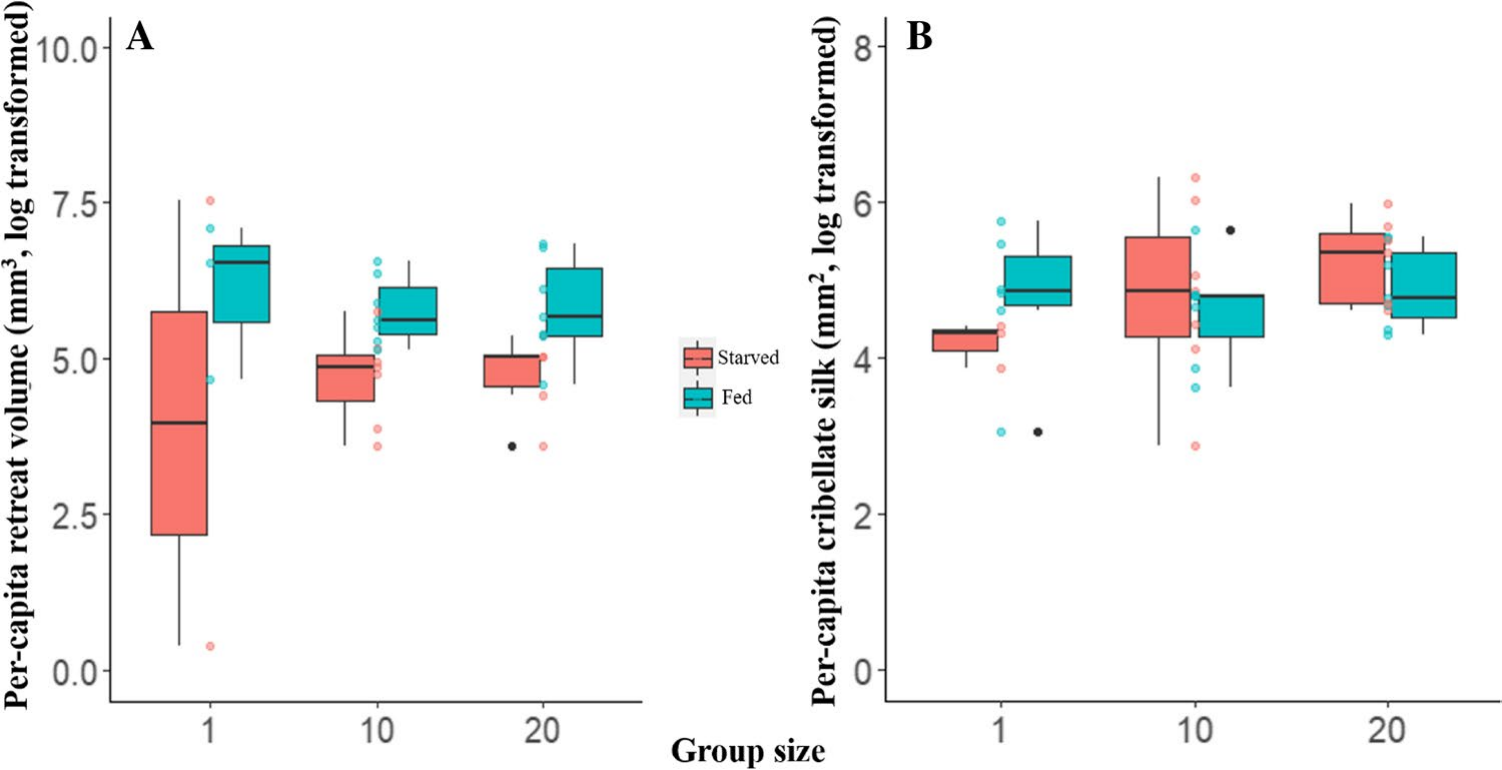
The influence of group size and food treatment on per-capita retreat volume (**A**) and cribellate capture silk area (**B**). Boxes represent lower and upper quartiles, whilst whiskers represent data outside the lower and upper quartiles. Internal horizontal lines represent median values. Filled circles in red and blue represent individual colonies. Red circles indicate colonies in the starved state, and blue circles indicate the same colonies in the well-fed state. Filled circles in black represent outliers

Group size did not have a significant effect on per-capita investment in retreat volume and cribellate silk area (Fig. 2, Table 1). Interestingly, solitary spiders and groups of 10 and 20 spiders showed similar per-capita investment in retreat and cribellate silk. Food treatment had a significant effect only on the retreat volume but not on the cribellate area (Fig. 2, Table 1). Across the three group sizes, well-fed colonies invested in larger per-capita retreats than starved colonies. However, well-fed and starved colonies showed similar investment in per-capita cribellate capture silk. Therefore, our results reveal a trade-off between investment in safety (retreat) and foraging (capture web), which is adjusted by the collective according to need.

**Table 1:** Estimates from two independent mixed models consisting of either per-capita retreat volume or cribellate capture silk area as the dependent variable

Dependent variable	Fixed effects	Estimate ± SD	Pairwise contrasts Estimates for fed-starved treatment
Retreat	Intercept	4.6 ± 0.7 (3.3 – 5.9) *	1.01 (0.67 – 1.35) *
	Group size 10	0.1 ± 0.8 (− 1.4 – 1.7)	
	Group size 20	0.3 ± 0.8 (− 1.3 – 1.8)	
	Fed treatment	1.0 ± 0.2 (0.7 – 1.4) *	
Cribellate silk	Intercept	4.7 ± 0.3 (4.1 – 5.4) *	− 0.15 (− 0.46 – 0.18)
	Group size 10	0 ± 0.4 (− 0.8 – 0.8)	
	Group size 20	0.4 ± 0.4 (− 0.4 – 1.2)	
	Fed treatment	− 0.1 ± 0.2 (− 0.5 – 0.2)	

95% credible intervals (CI) are shown within parenthesis. Significant values are highlighted by an asterisk (*).

## Discussion

The starvation-predation risk hypothesis posits that natural selection should favour those individuals that can assess the risk of predation and trade-off foraging gains with safety (Lima and Dill 1990). However, the adaptive role of extended phenotypes in facilitating such trade-offs is not well understood. Here, we showed that the architecture of the extended phenotypes itself can be subjected to trade-offs, reflecting the starvation-predation risk model. We found that hungry colonies of *S. sarasinorum* invested similarly in cribellate capture silk as well-fed colonies, whereas hungry colonies invested significantly less in retreats, showing a trade-off in the investment of retreats for capture webs. A previous study on *S. sarasinorum* demonstrated that group living did not entail per-capita savings in capture web silk (Beleyur et al. 2021). Here, we confirm this finding and additionally show that group living does not lead to savings on per-capita retreat silk investment, even when colonies were hungry. Our results suggest that simple proximate cues, such as hunger state, can enable groups of individuals to reach a consensus on which extended phenotypic architecture to channelize collective investment and when.

Social spiders are uncommon among arachnids, but they have evolved multiple times in independent lineages (Agnarsson et al. 2006; Johannesen et al. 2007). In every case, social evolution in spiders is characterised by low dispersal potential, high levels of inbreeding and high colony extinction rates (Lubin and Bilde 2007). Despite the low general dispersal potential which usually is the domain of spiderlings after hatching from their silken egg-sacs, social spiders can disperse solitarily as adults (mated females) and establish new colonies or groups of individuals leave their nest (group dispersal) to form adjacent satellite retreats that are interconnected to the parent colony by common capture webs (Bilde et al. 2007; Parthasarathy and Somanathan 2018). Our experiments mimic group dispersal because we generated experimental groups from field-collected colonies, manipulated their hunger levels and gave them a new environment to construct capture webs and retreat shelters. We speculate that well-fed spiders in the wild are more likely to establish new independent colonies or satellite retreats because well-fed experimental groups invested more in retreats. Giving priority to building retreat shelters can aid in survival in the wild by conferring improved antipredatory protection. Our observations on the web architecture spanned only for 3 days in this study, so it is impossible to assess whether starved colonies invest less in retreats in general or if they take longer to match the retreat sizes built by well-fed colonies. Field studies that test whether colonies with relatively smaller retreats suffer from greater predation would be desirable.

It is yet unclear how the relative investment in retreat size versus cribellate capture silk might shape collective foraging behaviours of social spider colonies. Previous studies have shown among-colony variation in latencies to capture prey (Keiser and Pruitt 2014). However, such among-colony differences in behaviour might be a property of the extended phenotype, in addition to, or instead of, the property exhibited by organisms per se. For instance, a smaller retreat size might simply enable spiders to emerge quickly and attack the trapped prey faster. Furthermore, communal prey capture behaviour can vary among independently derived social spider species (Grinsted et al. 2022), possibly because of among-species variation in the web phenotype. Such signalling functions of extended phenotypes are known in animals from other taxa (Moreno 2012; Schaedelin and Taborsky 2009). Therefore, the architecture of extended phenotypes should not be ignored in studies examining the behavioural variation of individuals and/or groups.

In conclusion, we show that internal states of the animal such as hunger can facilitate trade-offs in collective investment in extended phenotypes in *S. sarasinorum*. It is yet unclear how such architectural trade-offs can influence behavioural outcomes of colonies. Unlike active foragers, web-building sit-and-wait predators rely heavily on their extended phenotypes for foraging and protection, and therefore, further research is advocated to understand the selective benefits of the adaptive evolution of architectural phenotypes.
